# Combined physiological and transcriptome analysis revealed the response mechanism of *Pogostemon cablin* roots to p-hydroxybenzoic acid

**DOI:** 10.3389/fpls.2022.980745

**Published:** 2022-09-26

**Authors:** Wuping Yan, Shijia Cao, Xiaofeng Liu, Guanglong Yao, Jing Yu, Junfeng Zhang, Tengfei Bian, Wengang Yu, Yougen Wu

**Affiliations:** ^1^College of Tropical Crops, Hainan University, Haikou, China; ^2^College of Horticulture, Hainan University, Haikou, China; ^3^School of Agricultural Sciences, Jiangxi Agricultural University, Nanchang, China; ^4^Department of Medicinal Chemistry, University of Florida, Gainesville, FL, United States; ^5^Hainan Yazhou Bay Seed Laboratory, Sanya Nanfan Research Institute of Hainan University, Sanya, China

**Keywords:** *Pogostemon cablin* (patchouli), root, soil sickness, RNA-seq, p-hydroxybenzoic acid, active oxygen metabolism, autotoxicity

## Abstract

*Pogostemon cablin* (patchouli) cultivation is challenged by serious soil sickness, of which autotoxins accumulation is a major cause. p-hydroxybenzoic acid (p-HBA) is one of the main autotoxins of patchouli. However, the molecular mechanism underlying the response of patchouli to p-HBA remains unclear. In this study, RNA-sequencing combined with physiological analysis was used to monitor the dynamic transcriptomic and physiological changes in patchouli seedlings 0, 6, 12, 24, 48, and 96 h after p-HBA treatment. p-HBA stress inhibited root biomass accumulation, induced excessive hydrogen peroxide accumulation and lipid peroxidation, and activated most antioxidant enzymes. Compared with that of the control, the osmotic adjustment substance content was elevated with treatment. Subsequently, 15,532, 8,217, 8,946, 2,489, and 5,843 differentially expressed genes (DEGs) at 6, 12, 24, 48, and 96 h after p-HBA treatment, respectively, were identified in patchouli roots. GO functional enrichment analysis showed that the DEGs were enriched mainly in plasma membrane, defense response, response to chitin, DNA-binding transcription factor activity and abscisic acid-activated signaling pathway. The upregulated genes were involved in glycolysis/gluconeogenesis, cysteine and methionine metabolism, starch and sucrose metabolism, biosynthesis of unsaturated fatty acids, and linoleic acid metabolism. Genes associated with MAPK signaling pathway-plant, plant-pathogen interaction, plant hormone signal transduction were downregulated with p-HBA treatment. These pathways are related to root browning and rotting, leading to plant death.

## Introduction

*Pogostemon cablin* Benth. (patchouli), (Blanco) a member of the Lamiaceae (Labiatae) family, is a valuable aromatic plant that has been widely used in medicines and perfumes (Swamy and Sinniah, 2016; China Pharmacopoeia Committee, 2020). However, there are serious continuous cropping obstacles (also called soil sickness; Zhou et al., 2018) in the cultivation of patchouli, which greatly reduce its yield and quality (Xu et al., 2015; Zhang J. F. et al., 2018; Zeng et al., 2020; Yan et al., 2022). Many studies have shown that physical and chemical deterioration of rhizosphere soil, imbalances in the rhizosphere soil microbial community structure and autotoxicity are the main causes of soil sickness (Dong L. L. et al., 2018; Zhang B. et al., 2020). Autotoxicity refers to the phenomenon in which plants release allelochemicals to inhibit the growth and development of the same plant, which is a special type of allelopathy (Liu et al., 2019; Zhang Z. Z. et al., 2020). It mainly inhibits plant growth by changing the micro- and ultra-structure of cells, inhibiting cell division and elongation, disturbing the antioxidant system, increasing cell membrane permeability, interfering with the growth regulator system, affecting respiration and photosynthesis, inhibiting water and nutrient uptake, and affecting protein and nucleic acid synthesis and metabolism (Cheng and Cheng, 2015). In recent years, many researchers have isolated and identified allelochemicals, mainly including organic acids, phenolic acids, plant volatiles, terpenoids, coumarins, flavonoids, and monoterpenes, in different plants (Cheng and Cheng, 2015; Scavo et al., 2019). Among them, phenolic acids are the most studied and have strong activity and have become a research hotspot concerning soil sickness caused by allelopathy (Hao et al., 2010). In previous studies, various phenolic acids compounds, such as benzoic acid, cinnamic acid, vanillic acid, salicylic acid, p-hydroxybenzoic acid (p-HBA), and tetradecanoic acid, were isolated and identified from patchouli plants and their rhizosphere soils (Wu et al., 2013). It was found that p-HBA had an obvious autotoxic effect on the growth of patchouli (Xu et al., 2015).

p-HBA is one of the main phenolic acids in the replant soil of many plants and is an important factor causing soil sickness (Huang et al., 2020; Wu et al., 2021). p-HBA can directly regulate the activities of metabolic enzymes in the glycolysis and pentose phosphate oxidation pathways, which results in inhibition of seed germination and root growth (Muscolo et al., 2001; Lim et al., 2002). Weir et al. (2004) and Zhang et al. (2010) showed that p-HBA not only hindered the carbon and nitrogen metabolism of plants, but also damaged the DNA and protein of plants. Huang et al. (2020) showed that p-HBA treatment decreased the expression of cell cycle-related genes and the length of mature root cells in cucumber roots. Liang et al. (2021) showed that p-HBA treatment induced changes in photosynthesis, respiration and reactive oxygen species-related genes in *Populus* × *euramericana* “Neva” leaves.

Plant roots are the first organs to perceive the soil environment, and their architectural plasticity is considered the main toolbox for plants to face and adapt to oscillating environmental conditions (Zhang W. et al., 2018). As plant roots are directly exposed to soil allelochemicals, the root growth defect may be the primary and potentially decisive phytotoxic response to allelochemical stress. The analysis of root morphological changes and the related physiological and biochemical metabolic processes and gene expression is an important means to clarify the mechanisms underlying autotoxicity (Soltys et al., 2014). Therefore, the study of root autotoxicity must be given special attention, but this has often been ignored in previous studies. In view of the lack of research on autotoxicity in patchouli, this study revealed the response mechanism of patchouli roots to the autotoxicity of p-HBA by physiological analysis combined with transcriptome sequencing.

## Materials and methods

### Plant materials and p-hydroxybenzoic acid treatment

The cultivated patchouli variety Naixiang obtained from the patchouli germplasm resource garden of Hainan University (Haikou, China) was used for the experiments. Healthy, young branches of patchouli with uniform growth were selected for cutting propagation in a sterilized sand bed (Yan et al., 2021). At 30 days after cutting, the cutting seedlings of patchouli with vigorous and consistent root growth were selected, transplanted into sterilized silica sand and placed in a plastic square basin (40 cm × 20 cm × 15 cm) at a density of 15 plants/pot, placed in a greenhouse, under 15,000 LX light intensity for 14 h/day, darkness for 10 h/day, a temperature of 25°C, and 60% relative humidity to control soil humidity, temperature, fertility, pathogens and other unpredictable factors that may affect the severity of disease and the growth of pathogens. Hoagland nutrient solution was used to provide essential nutrients for the normal growth of patchouli plants. After 30 days of culture, Hoagland nutrient solution containing 1 mmol/L p-HBA was used for subsequent p-HBA stress treatment. The pretest showed that 1 mmol/L p-HBA caused obvious autotoxic symptoms in patchouli seedlings, but it was not enough to kill the plants. Under the same conditions, Hoagland nutrient solution without p-hydroxybenzoic acid was used as a blank control. At 0, 6, 12, 24, 48, and 96 h after treatment, root samples of patchouli seedlings were taken, washed with sterile water, and divided into two parts. One part was frozen in liquid nitrogen and stored at –80°C for subsequent use, and the other part was placed on ice for physiological and biochemical index measurements. The samples at each time point from the roots of five independent patchouli plants were mixed. There were 3 biological replicates at each time point, and a total of 18 root samples were obtained (p-HBA_0 h_root, p-HBA_6 h_root, p-HBA_12 h_root, p-HBA_24 h_root, p-HBA_48 h_root and p-HBA_96 h_root, each with 3 biological replicates).

### Determination of physiological and biochemical traits

Fresh root samples of patchouli seedlings treated with p-HBA for different times were used as materials for the determination of antioxidant enzyme activity, phenylalanine aminotransferase (PAL) activity, H_2_O_2_ content, O_2_^–^ content, malondialdehyde (MDA) content and osmoregulatory substance content. Superoxide dismutase (SOD) activity, peroxidase (POD) activity, catalase (CAT) activity, and MDA content were analyzed using the WST-8, guaiacol oxidation, ammonium molybdate and thiobarbituric acid methods, respectively, with the corresponding kits (SOD-2-W, POD-2-Y, CAT-2-W, and MDA-2-Y). The content of soluble protein (SPR) and proline (Pro) were measured by Coomassie brilliant blue staining and the acidic ninhydrin method with the corresponding kits (KMSP-2-W and PRO-2-Y). The contents of O_2_^–^ and H_2_O_2_ were measured according to the hydroxylamine hydrochloride reaction and titanous sulfate method described by Li and Yi (2012) with the corresponding kits (SA-2-G and H_2_O_2_-2-Y). PAL catalyzes the cleavage of L-phenylalanine to trans-cinnamic acid and ammonia, and the maximum absorption of trans-cinnamic acid occurs at 290 nm. PAL activity was studied using the trans-cinnamic acid method with PAL-2-Y. All kits and reagents were purchased from Suzhou Keming Biotechnology Co., Ltd (Suzhou, China), and all test procedures were carried out in accordance with the manufacturers’ instructions. The detection of each index was repeated three times.

### RNA isolation, library construction, and sequencing

Using fresh root samples treated with p-HBA for different times as materials, total RNA was extracted by a mirvana miRNA Isolation Kit (Ambion) according to the manufacturer’s guidelines. An Agilent 2100 Bioanalyzer (Agilent Technologies, Santa Clara, CA, United States) was used to evaluate RNA integrity, and samples with an RNA integrity number (RIN) ≥ 7 were selected for subsequent analysis. Next, a total of 18 cDNA libraries were constructed according to the manufacturer’s instructions using a TruSeq Stranded mRNA Sample Prep Kit (Illumina, San Diego, CA, United States). Finally, these libraries were sequenced on the Illumina HiSeq × ten platform, and 150 bp paired terminal reads were generated by OE Biotech Co., Ltd. (Shanghai, China).

### Bioinformatics analysis of transcriptome sequencing data

The raw data obtained by transcriptome sequencing were processed using Trimmomatic (Bolger et al., 2014) to remove poly N and low-quality reads to obtain clean reads. Clean reads were mapped to the patchouli reference genome using hisat2 (Kim et al., 2015). Htseq count (Anders et al., 2015) was used to estimate the read counts of each gene, and Cufflinks was used to calculate the transcripts per thousand bases and the reads per million mapped reads (FPKM, Trapnell et al., 2010). The DESeq R software package (Anders and Huber, 2012) was used to analyze the differential expression between groups using read counts. The thresholds of significant differential expression were set as | log2-fold change| ≥ 1 and *p*-value ≤ 0.05. To verify the expression patterns of genes in different groups and samples, hierarchical cluster analysis was carried out on differentially expressed genes (DEGs). To further evaluate the biological function of these DEGs, GO and KEGG enrichment analyses were performed using the goseq R software package (Young et al., 2010) and Kobas 2.0.12 software (Mao et al., 2005).

### Validation of differentially expressed genes by quantitative real-time PCR

The total RNA used to construct the RNA-seq library was used for quantitative real-time PCR (qRT-PCR). cDNA was synthesized using MonScript™ RTIII All-in-One Mix with dsDNase (Monad Biotech Co., Ltd., Shanghai, China) according to the manufacturer’s instructions. Twenty DEGs were randomly selected for qRT–PCR analysis. First, qRT–PCR primers were designed using Primer Premier 5.0 (Premier Biosoft International, Palo Alto, CA, United States), and 20 DEGs are listed in Supplementary Table 1. These qRT–PCR primers were synthesized by Taihe Biotechnology Co., Ltd. (Beijing, China). Then, the qRT–PCR experiment was performed using the MonAmp™ ChemoHS qPCR Mix (Monad Biotech Co., Ltd., Shanghai, China) kit according to the kit instructions. The qRT–PCR experimental system was as follows: the total volume was 20 μL, including 10 μL MonAmp™ ChemoHS qPCR Mix, 0.4 μL forward and 0.4 μL reverse primers (final concentration: 0.2 μM), 8.7 μL sterile deionized water, and 0.5 μL cDNA. qRT–PCR analysis was performed on a LightCycler^®^ 96 system (Roche, Switzerland), and each experiment included three technical replicates. The 18S ribosomal RNA (18S rRNA) gene was used as the internal control for qRT-PCR because the expression level of the gene is relatively stable in different tissues and samples at different time points (Yan et al., 2021). The relative expression levels of each gene were calculated using the 2^–ΔΔ^ ^Ct^ method (Livak and Schmittgen, 2001).

### Statistical analysis

Patchouli roots with the same treatment times were taken from different pots as biological replicates. The whole experiment was repeated at least three times. For root fresh weight indicators, each data point was an average of at least five individual measurements. For other biochemical indicators, each data point was the average of at least three individual measurements. All physiological and biochemical data are included in the Excel software (Microsoft, United States) for Microsoft Office 2016. The experimental results are presented as the mean ± standard deviation (mean ± SD). One-way ANOVA was performed using IBM SPSS Statistics 20.0 software (IBM, United States). The significance of differences between means was determined by the least significant difference (LSD) test at the 0.05 probability level, and the significance values are denoted with different lowercase letters. Bioinformatics analysis of transcriptome data was performed on the free online oebiotech cloud platform.^1^

## Results

### Phenotypic and physiological responses of patchouli roots to p-hydroxybenzoic acid

**FIGURE 1:**
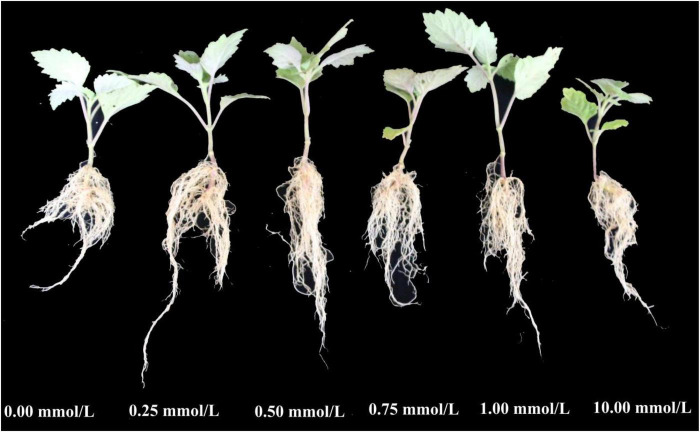
Phenotypic characteristics of patchouli seedlings treated with different concentrations of p-hydroxybenzoic acid for 12 h.

**FIGURE 2:**
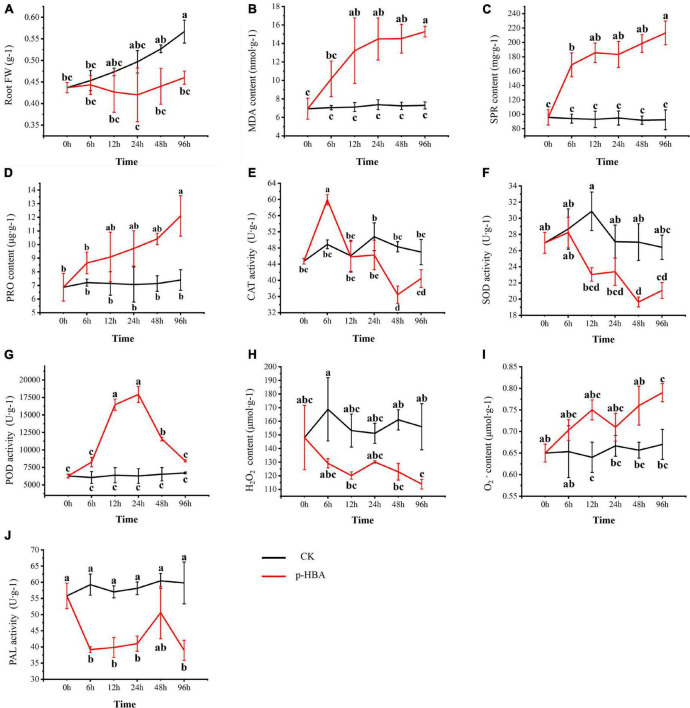
Physiological characteristics of patchouli seedlings treated with 1 mmol/L p-hydroxybenzoic acid for 0, 6, 12, 24, 48, and 96 h. **(A)** The root fresh weight. **(B)** Malonaldehyde. **(C)** Soluble protein content. **(D)** Proline content. **(E)** Catalase activity. **(F)** Superoxide dismutase activity. **(G)** Peroxidase activity. **(H)** H_2_O_2_ content. **(I)** O_2_ content. **(J)** Phenylalanine ammonia-lyase. Root FW, Root fresh weight; MDA, Malonaldehyde; SPR, Soluble Protein; PRO, Proline; CAT, Catalase; SOD, Superoxide Dismutase; POD, Peroxidase; PAL, Phenylalanine Ammonia-Lyase.

Treatment with 1 mmol/l p-HBA significantly inhibited the root growth of patchouli seedlings (Figures 1, 2). The root fresh weight of the treatment group was lower than that of the control group in the same period at all stages. The root fresh weight index of the treatment group was significantly inhibited at 96 h of p-HBA treatment (*p* < 0.05), being 18.83% lower than that of the control group in the same period. The contents of MDA, soluble protein and proline in roots increased with increasing treatment time under p-HBA treatment. After 6 h of p-HBA treatment, the content of soluble protein was significantly higher than that of the control group (*p* < 0.05), and the content of MDA and Pro in roots was higher than that of the control group, but there was no significant difference (*p* > 0.05). At 12, 24, and 48 h, the contents of MDA and soluble protein in roots were significantly higher than those in the control group at the same time (*p* < 0.05), and the content of Pro was higher than that in the control group, but the difference was not significant (*p* > 0.05). After 96 h of treatment, the contents of MDA, soluble protein and proline in roots reached the highest levels, which were significantly increased by 109.27, 120.86, and 63.59%, respectively, compared with the control group at the same period (*p* < 0.05).

Under the treatment of 1 mmol/l p-HBA, there were some differences in the change trends of different antioxidant enzyme activities in the roots of patchouli seedlings. Under the treatment of p-HBA, CAT activity first increased and then decreased with the increase of treatment time, peaking at 6 h, and was significantly increased by 22.53% compared with the control group at the same period (*p* < 0.05) but was lower than the control group at 24, 48, and 96 h. Under the treatment of p-HBA, the activity of SOD decreased gradually with increasing treatment time and reached the lowest level at 48 h, which was significantly lower than that of the control group at the same period by 27.38% (*p* < 0.05). The activity of POD increased under the treatment of p-HBA and peaked at 24 h, being 185.93% higher than that of the control in the same period (*p* < 0.05). At the same time, the ROS content of the *Pogostemon* root system was measured. It was found that the content of superoxide anion decreased significantly, and hydrogen peroxide accumulated significantly under the treatment of p-HBA. After 96 h of treatment, the superoxide anion content decreased by 27.04%, and the hydrogen peroxide content increased by 17.91% (*p* < 0.05).

Root PAL activity was significantly inhibited by p-HBA treatment. At 48 h after treatment, PAL activity in the treatment group decreased compared with that in the control group, but the difference was not statistically significant (*P* > 0.05). After 96 h of treatment, the PAL activity of the treatment group reached the lowest level, which was significantly decreased by 34.85% compared with that of the control group (*p* < 0.05).

The statistical correlation analysis of different physiological indexes showed that CAT and SOD were positively correlated with each other in this experiment and that both were significantly negatively correlated with POD, MDA and H_2_O_2_ (Supplementary Table 2). There were significant negative correlations between both SPR and POD and root fresh weight. Under p-HBA stress, with increasing stress time, CAT and SOD activity decreased and POD activity increased, resulting in a decrease in H_2_O_2_ scavenging capacity, intensification of root oxidative stress, and inhibition of root growth.

### Dynamic transcriptomic profiles of patchouli roots in response to p-hydroxybenzoic acid

To study the molecular response of patchouli seedlings to p-HBA stress, patchouli seedlings were treated with 1 mmol/L p-HBA solution. Fresh roots were collected and sequenced at 0, 6, 12, 24, 48, and 96 h after treatment. A total of 18 cDNA libraries were constructed and sequenced, and the original reads and clean reads are shown in Supplementary Table 3. The original reads of sequencing were between 34.52 and 40.85 M. After removing the adapter sequence, low-quality reads and reads containing more than 5% N bases, clean reads were obtained between 34.27 and 40.55 M. The average valid bases, Q30 bases and GC percentages were 98.07, 95.04, and 46.87%, respectively. On average, 99.96% of clean reads were mapped to the patchouli genome. Transcriptome characteristics of 18 samples were compared intuitively by correlation heatmap and principal component analysis (PCA) diagram (Supplementary Figure 1). The results showed that there was a high correlation among the three biological replicates, and the samples at different time points were separated to some extent, indicating that p-hydroxybenzoic acid drove the gene expression of patchouli roots. All raw data were deposited at the NCBI Sequence Read Archive (SRA) under the accession number PRJNA850618.

### Identification of differentially expressed genes in response to p-hydroxybenzoic acid stress

**FIGURE 3:**
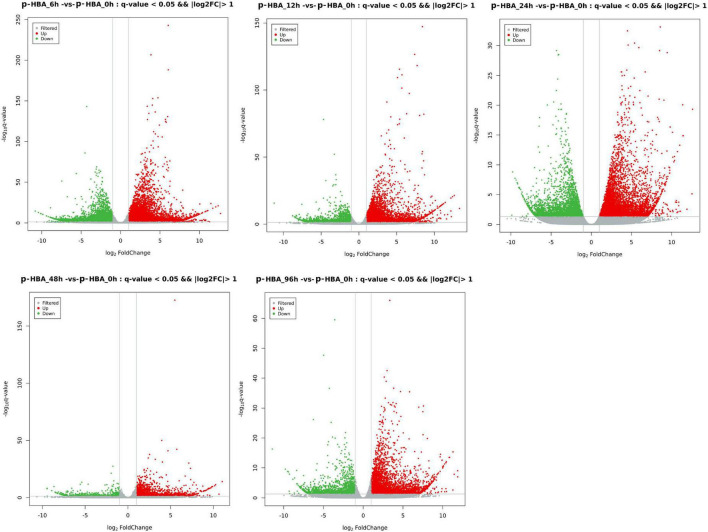
Volcano plot for differentially expressed genes in patchouli roots in response to p-hydroxybenzoic acid stress. The red dots indicate upregulated genes, the green dots indicate downregulated genes, and the gray dots indicate genes with no significant difference in expression.

**FIGURE 4:**
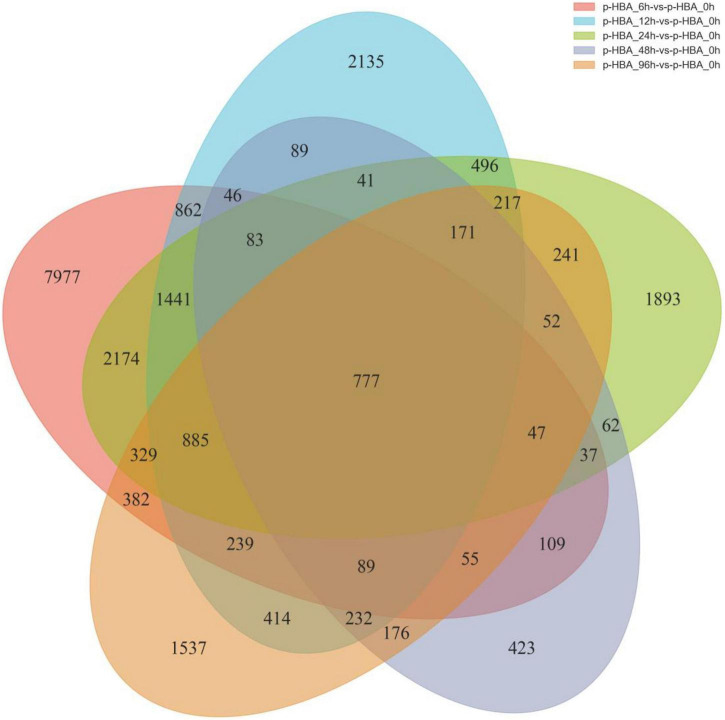
Venn diagram of the differentially expressed genes in patchouli roots in response to p-hydroxybenzoic acid stress.

To identify the potential p-HBA stress response genes in patchouli roots, FPKM values were used to calculate the expression level of a gene from the p-HBA-treated compared to the unstressed samples (Supplementary Figure 2). Based on a threshold | log2Fold Change | > 1 with *q*-value < 0.05, we identified a total of 23,711 DEGs in the five comparison groups. Compared with those at 0 h, 9,049, 5,843, 5,521, 1,843, and 4,082 DEGs were upregulated, and 6,483, 2,374, 3,425, 646, and 1,761 DEGs were downregulated at 6, 12, 24, 48, and 96 h, respectively. The average proportion of upregulated and downregulated genes in each comparison group was 67.00 and 33.00%, respectively, indicating that p-hydroxybenzoic acid stress significantly activated the expression of more genes in patchouli roots (Figure 3). We constructed a Venn diagram to analyze the specific and overlapping DEGs in each comparison group (Figure 4 and Supplementary Table 4). As shown, 777 DEGs were coexpressed in the five comparisons, indicating that these genes were constitutively involved in the p-HBA stress response. A total of 7,977, 2,135, 1,893, 423, and 1,537 DEGs were specifically expressed after p-HBA treatment for 6, 12, 24, 48, and 96 h, and these genes may play specific roles in the p-HBA response during different periods.

### GO and KEGG enrichment analysis of differentially expressed genes

**FIGURE 5:**
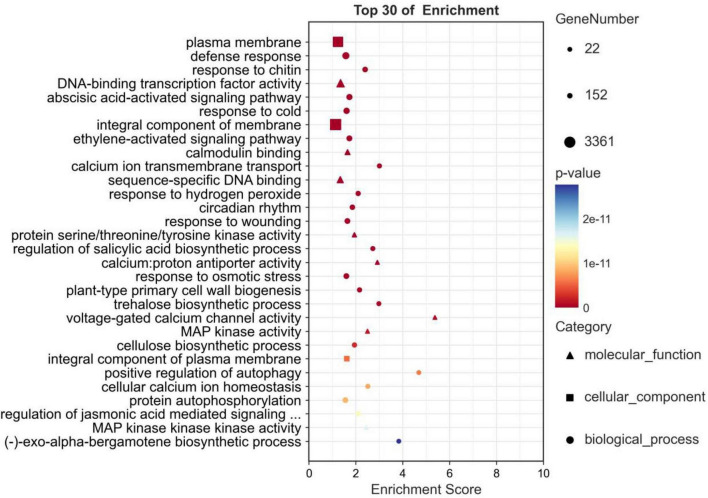
Go enrichment analysis of the DEGs in p-hydroxybenzoic acid-treated root tissues compared to controls.

GO enrichment analysis was performed on DEGs in five comparison groups (HBA_6 h-vs.-HBA_0 h, HBA_12 h-vs.-HBA_0 h, HBA_24 h-vs.-HBA_0 h, HBA_48 h-vs.-HBA_0 h, HBA_96 h-vs.-HBA_0 h) to clarify their specific biological functions. A total of 23,711 DEGs were obtained, of which 13,028 DEGs were annotated in the biological process (BP), 14,308 DEGs in the cellular component (CC), and 13,965 DEGs in the molecular function (MF) categories. In BPs, there were 490 terms with a *p*-value ≤ 0.05 and 312 terms with an FDR ≤ 0.05; in CCs, there were 69 terms with a *p*-value ≤ 0.05 and 32 terms with an FDR ≤ 0.05; in MFs, there were 325 terms with a *p*-value ≤ 0.05 and 181 terms with an FDR ≤ 0.05. There were 884 terms with *p*-values ≤ 0.05, and the first three terms with minimum *p*-values were plasma membrane (GO:0005886, *p*-value: 5.4e-35); defense response (GO: 0006952, *p*-value: 1.9e-32); and response to chitin (GO: 0010200, *p*-value: 1.2e-31) (Figure 5). A total of 525 terms had an FDR ≤ 0.05.

**FIGURE 6:**
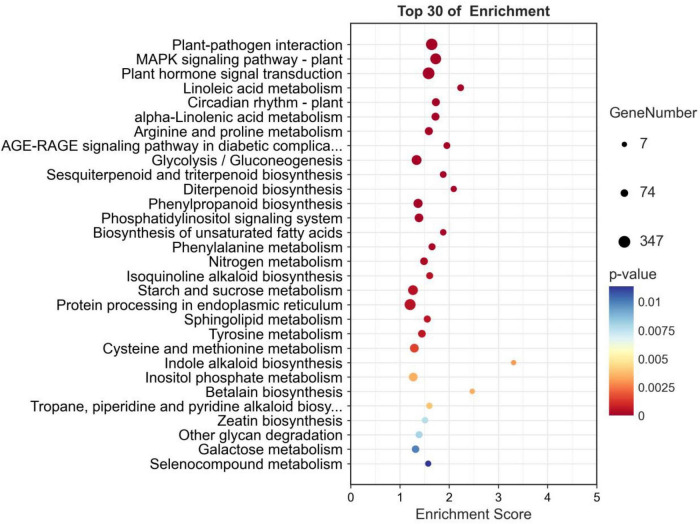
KEGG pathway enrichment analysis of the DEGs in p-hydroxybenzoic acid-treated root tissues compared to controls.

Pathway analysis of DEGs can help to obtain the pathways related to DEGs to infer which pathway changes may be related to p-HBA stress. A total of 23,711 DEGs were identified, and 4,870 DEGs were annotated to KEGG pathways. There were 37 pathways with *p*-values ≤ 0.05, and the first three pathways with minimum *p*-values were plant–pathogen interaction (ko04626, *p*-value: 1.2e-19); MAPK signaling pathway—plant (ko04016, *p*-value: 2.2e-19); and plant hormone signal transduction (ko04075, *p*-value: 1.1e-18) (Figure 6). A total of 30 pathways had an FDR ≤ 0.05.

### Identification of key genes in response to p-hydroxybenzoic acid stress

Combined with transcriptome data and relevant literature, the DEGs related to alcohol dehydrogenase, pyruvate decarboxylase and thiamine pyrophosphate requiring enzymes in the glycolysis/gluconeogenesis pathway were screened. Thirty-four, 30, 21, 13, and 20 related DEGs were screened at 6, 12, 24, 48, and 96 h of allelochemical p-HBA stress treatment. Among them, 32, 29, 21, 13, and 20 were upregulated, and 2, 1, 0, 0, and 0 were downregulated. Under the stress of the allelochemical p-HBA, the DEGs related to ethanol dehydrogenase, pyruvate decarboxylase and thiamine pyrophosphatase in the roots of patchouli were mainly upregulated, and the proportion increased with increasing treatment time. The DEGs related to abscisic acid receptor, chloroplast copper transporting ATPase PAA2, mitogen activated protein kinase and protein phosphatase in the plant MAPK signal transduction pathway were screened. At 6, 12, 24, 48, and 96 h of allelochemical p-HBA stress treatment, 72, 36, 46, 11, and 23 related DEGs were screened out, of which 44, 24, 33, 8, and 16 were upregulated and 28, 12, 13, 3, and 7 were downregulated. The root abscisic acid receptor, chloroplast copper transport ATPase-, mitotic activated protein kinase- and protein phosphatase-related DEGs in patchouli were mainly upregulated under stress imposed by the allelochemical p-HBA.

Ca^2+^/calmodulin-dependent protein kinase (EF hand protein superfamily), calcium-dependent protein kinase (calcium-dependent protein kinase), disease resistance protein RPM1 (disease resistance protein RPM1), and serine/threonine protein kinase pbs1 (serine/threonine protein kinase) WRKY proteins were screened for DEGs. Forty, 32, 30, 11, and 28 related DEGs were screened at 6, 12, 24, 48, and 96 h of allelochemical p-HBA stress treatment, including 28, 26, 20, 9, and 24 upregulated and 12, 6, 10, 2, and 4 downregulated. It can be seen that under p-HBA stress, Ca^2+^/calmodulin dependent EF hand protein kinase, calcium dependent protein kinase, disease resistance protein RPM1 like, serine/threonine protein kinase pbs1 and WRKY protein-related DEGs in patchouli roots were mainly upregulated, among which Ca^2+^/calmodulin dependent EF hand protein kinase and calcium dependent protein kinase-related DEGs were mainly upregulated. The number of differential genes was 0 at 48 and 96 h. The DEGs related to the resistance protein RPM1 were downregulated.

The DEGs related to aldehyde dehydrogenase, methyltransferase, cinnamate-4-hydroxylase, peroxidase and phenylalanine ammonia lyase in the phenylpropane biosynthesis pathway were screened. At 6, 12, 24, 48, and 96 h of p-HBA stress treatment, 25, 15, 17, 2, and 15 related DEGs were screened, of which 11, 9, 2, 1, and 0 were upregulated and 14, 6, 15, 1, and 15 were downregulated. Under p-HBA stress, the DEGs related to ethanol dehydrogenase, cinnamate carboxylase, peroxidase and phenylalanine aminolyase in the roots of patchouli were downregulated, and a large proportion of ethanol dehydrogenase-related DEGs was downregulated at 48 and 96 h of treatment. Phenylalanine ammonia lyase-related DEGs were downregulated.

### Quantitative real-time PCR verification results

**FIGURE 7:**
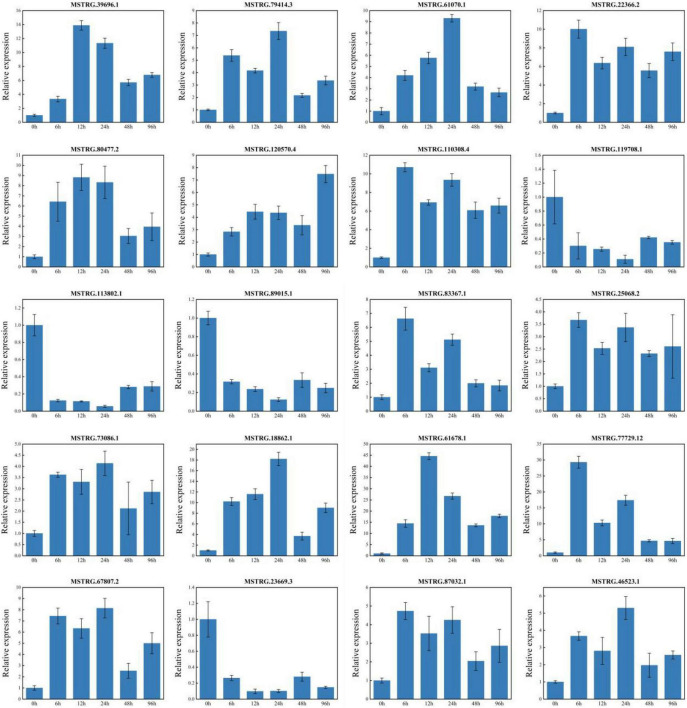
qRT-PCR verification of differentially expressed genes.

To verify the accuracy of the RNA-seq data, the 20 DEGs in the roots of patchouli under p-HBA stress for 6, 12, 24, 48, and 96 h, including DEGs related to plant-pathogen interaction, MAPK signaling pathway—plant, phenylpropanoid biosynthesis, and plant hormone signal transduction, were selected (Figure 7). Linear regression analysis showed that the RT-PCR data were significantly correlated with the RNA-seq data (between 0.6244 and 0.9889, with an average of 0.9231), indicating that the qRT-PCR results for the DEGs were consistent with the sequencing results. This consistency indicates that the RNA-seq data in this study were reliable.

## Discussion

### Physiological response of patchouli roots under p-hydroxybenzoic acid stress

Soil sickness is complex problem caused by the interaction of plants, root exudates, the rhizosphere soil environment and other factors (Zhang B. et al., 2020). Studies have shown that allelopathy is one of the main factors causing soil sickness in patchouli (Xu et al., 2015). p-Hydroxybenzoic acid is an allelochemical with strong autotoxicity potential, which has been reported in studies of cucumber (Huang et al., 2020) and grape (Liu et al., 2019). In this study, we found that p-hydroxybenzoic acid could inhibit the accumulation of root biomass in *Pogostemon cablin* and lead to abnormal changes in root ROS, protective enzyme systems and osmotic adjustment substances.

O^2–^ and H_2_O_2_ are important ROS in plants. Excessive accumulation of these compounds in plants will lead to oxidative stress, damage cell integrity, and then affect growth and development (Cheng and Cheng, 2015; Zhang et al., 2016; Xin et al., 2019). In this study, the H_2_O_2_ content in the roots of *Pogostemon cablin* was significantly higher than that in the control at the same period, and the O^2–^ content showed the opposite changes. The results showed that H_2_O_2_ was the main factor causing oxidative damage in the allelopathy of patchouli roots induced by p-HBA, which was consistent with research on the allelopathy of *Angelica sinensis* roots (Xin et al., 2019). Exogenous application of p-HBA could affect the activities of POD, SOD and CAT related to ROS scavenging and the expression of related genes (Zhang et al., 2012). In this study, combined with transcriptome data, the expression of genes related to antioxidant enzymes such as CAT and SOD was downregulated, and the activity of related enzymes was inhibited. POD activity was higher than that of the control during the whole period, the expression of related genes was upregulated, and many genes were enriched in the phenylpropanoid biosynthesis pathway. Similar results were found in studies of tobacco (Wang et al., 2022) and wolfberry (Mo et al., 2022) under stress. Taken together, POD plays an important role in the mechanism by which *Pogostemon cablin* roots respond to p-HBA stress.

Under the treatment of high concentrations of phenolic acids, the induced damage exceeds the self-protection ability of plants, resulting in the accumulation of harmful substances (Romagni et al., 2004). MDA is the final product of plant membrane peroxidation and an important indicator for evaluating cell homeostasis. In this study, it was found that the contents of propylene glycol, soluble protein and proline in the roots of patchouli increased significantly, suggesting that p-HBA led to oxidative stress in root cells of patchouli and destroyed cell homeostasis. At the same time, patchouli maintained the balance of osmotic pressure inside and outside the cell membrane through the accumulation of osmotic adjustment substances and actively protected the normal physiological process of plants (Singh et al., 2016; Jespersen et al., 2017).

Correlation analysis showed that CAT was positively correlated with SOD and negatively correlated with POD. Under normal circumstances, free radical production and elimination processes are active in plant cells. In this study, only SOD, CAT, POD were coordinated to maintain a low level of biological free radicals, thereby preventing free radical toxicity. Therefore, under p-HBA stress, CAT and SOD gradually decreased, and their ability to scavenge free radicals decreased, resulting in free radical toxicity in patchouli roots and inhibition of root growth.

### Specific gene expression patterns in patchouli roots driven by p-hydroxybenzoic acid stress

In current studies, allelopathy caused by allelochemicals is often regarded as an abiotic stress treatment. After exogenous treatment of plants, transcriptome-related technologies can help researchers analyze genes and provide a technical means for revealing the molecular response mechanism of plants to allelopathy by screening DEGs. Xiao et al. (2020) used exogenous phenolic acid treatment to study the allelopathy of cucumber. DEGs were enriched in plant hormone signal transduction, MAPK signaling pathway-plant, phenylalanine metabolism and plant pathogen interactions under cinnamic acid treatment. Dong C. et al. (2018) found that phenylpropanoid biosynthesis, flavonoid biosynthesis, metabolic pathways and plant MAPK signaling pathways were significantly activated in the roots of *Nelumbo nucifera*. In this study, it was found that the DEGs of patchouli were significantly enriched in GO terms such as metabolic process, stress response, transporter activity, structural and molecular activity. DEGs were significantly upregulated in the glycolysis/gluconeogenesis pathway, cysteine and methionine metabolism, starch and sucrose metabolism, unsaturated fatty acid biosynthesis pathway, and linolenic acid metabolism-related KEGG pathways. They were significantly downregulated in the plant MAPK signaling pathway, plant pathogen interaction, and plant hormone signal transduction-related pathways.

Carbohydrate metabolism is a basic element in maintaining the normal growth and development of plants. Researchers have found that, under stress, DEGs related to glycolysis/gluconeogenesis pathways in carbohydrate metabolism are significantly enriched. An et al. (2016) found that the key enzyme pyruvate decarboxylase gene in the glycolysis/gluconeogenesis pathway was significantly enriched in ramie under drought stress and analyzed the expression levels of protein-coding genes related to the glycolysis/gluconeogenesis pathway, indicating that plants resist abiotic stress by regulating available energy. Ethanol dehydrogenase is an important alcohol dehydrogenase that plays an important role in the plant response to stress. Xu et al. (2014) found that the transcription of genes related to ethanol dehydrogenase and pyruvate decarboxylase was significantly upregulated under waterlogging treatment. Combined with the transcriptome data in this study, it was found that the expression of genes such as ethanol dehydrogenase, pyruvate decarboxylase and thiamine pyrophosphatase was significantly upregulated, indicating that patchouli could mobilize available energy and actively respond to abiotic stress caused by the exogenous allelopathic autotoxicant p-HBA through the high expression of genes related to glycolysis/gluconeogenesis.

MAPK is a widely existing signal transduction pathway in plants. MAPK can phosphorylate a variety of downstream substrates, such as protein kinases, transcription factors and cytoskeletal proteins. Finally, the extracellular signal is gradually transmitted to the cells to regulate cell growth, differentiation, apoptosis and other stress responses to external stimuli (Ren et al., 2008; Gu et al., 2020). In *Arabidopsis thaliana*, abiotic stresses such as low temperature, drought, high osmotic pressure, high salt and mechanical damage could significantly increase the transcription level of AIMEKK1 (Mizoguchi et al., 1996) and could also activate the transient expression of AtMPK4 and AtMPK6 (Ichimura et al., 2000). It has also been reported that the signal module composed of the AtMPK1 gene and its upstream interaction gene MKK3 could phosphorylate and activate ROP-binding protein kinase, affecting the cell division of roots and further affecting the growth of roots (Enders et al., 2017). In rice, the OsMPK4, OsMPK5, OsMPK7, OsMPK8, OsMPK12, OsMPK14, and OsMPK15 genes could be induced by abiotic stresses such as drought, salt and temperature (Jeong et al., 2006; Reyna and Yang, 2006). Maize ZmMPK7 could be induced by ABA and H_2_O_2_ and could respond to various abiotic stresses, such as low temperature and drought (Zong et al., 2009). Treatment with the allelochemical p- HBA significantly downregulated the expression of genes related to the MAPK signal transduction pathway in the roots of patchouli, among which abscisic acid receptor, chloroplast copper transport ATPase, mitogen-activated protein kinase and protein phosphatase were downregulated. Abscisic acid is an important plant hormone and plays an important role in regulating plant growth and stress response. The abscisic acid receptor is a regulatory factor in the ABA signal transduction pathway in plants. Inhibiting protein phosphorylation promotes gene expression related to stress and improves the ability of plants to resist stress (Fujii et al., 2009). Researchers overexpressed ABA receptor-related genes in rice, and the results showed that its abundance significantly improved the ability of rice to resist drought stress and increased yield (Wang et al., 2019). Chen et al. (2017) found that the expression of ABA receptor-related genes in cotton was downregulated under drought conditions. Through a gene overexpression test, it was found that ABA receptor-related genes were involved in the plant response to drought/osmotic stress and defense. Combined with the transcriptome data in this study, it was found that the exogenous allelochemical p-HBA could reduce the expression of ABA receptor-related genes in the roots of patchouli, weaken the adaptability of plants to stressful environments, and affect the growth and development of patchouli.

The complexity of abiotic stress leads to complex and diverse components and mechanisms of signal perception and signal transduction. As a multifunctional secondary messenger, Ca^2+^ plays an extremely important role in almost all abiotic stress responses. For a particular abiotic stress, Ca^2+^ is not only involved in signal perception in stress but also ensures subsequent signal transmission (Dong et al., 2022). Chi et al. (2014) found that the Ca^2+^ content in rice roots increased significantly under exogenous ferulic acid stress. Calcium-dependent protein kinase is a kind of serine/threonine protein kinase that is usually composed of four core domains: the N-terminal variable domain, protein kinase domain, self-inhibitory domain and C-terminal calmodulin-like domain. Plants perceive environmental stimuli and transmit extracellular signals to cells through typical mechanisms, causing a series of reactions. Ca^2+^/calmodulin-dependent EF-Hand protein kinase can cause changes in the spatial structure of calcium-dependent protein kinase, release its own inhibitory domain, promote the recovery of protein kinase activity, phosphorylate downstream regulatory factors, and transmit the signal of the downstream regulatory network. Stress is one of the factors that induce gene expression of calcium-dependent protein kinases (CDPKs). Studies have shown that CDPKs are involved in regulating the response of various plants to environmental stress. Various environmental factors, such as drought and salt injury, can cause the differential expression of calcium-dependent protein kinase genes and the specific accumulation of their mRNAs (Li et al., 2019; Crizel et al., 2020; Shi and Zhu, 2022). Combined with the transcriptome data in this study, it was found that the Ca^2+^/calmodulin-dependent EF-Hand protein kinase and calcium-dependent protein kinase-related genes were highly expressed in the roots of patchouli under the allelopathic effects of p-HBA, positively responding to this stress. Similarly, CaM and CDPK genes were highly expressed in roots of replant patchouli. In summary, we speculated that Ca^2+^ signaling in the roots of patchouli was rapidly activated under p-HBA stress, the upstream molecules of the MAPK signaling pathway, activating the antioxidant defense system in ABA signaling and responding to p-HBA stress.

Phenylpropanoid metabolism is one of the most important secondary metabolic pathways in plants, producing more than 8,000 metabolites, and it plays an important role in plant growth and environmental interactions. At present, terrestrial plants have evolved a variety of branch pathways of phenylpropanoid metabolism, producing flavonoids, lignin, lignan, cinnamic acid amide and other metabolites (Dong and Lin, 2021). These phenylpropanoids have significant effects on many important traits of plants, affecting growth and development and responses to extreme environmental stresses (Liu et al., 2014). The phenylpropanoid biosynthesis pathway is the main pathway for the generation of phenylpropanoid compounds, which has been widely reported in the study of plant abiotic stress (Cao et al., 2020; Zhu et al., 2021). PAL is a gateway enzyme in the general phenylalanine metabolic pathway that catalyzes the production of trans-cinnamic acid from phenylalanine and guides the metabolic flux from the shikimate pathway to multifarious branches of phenylalanine metabolism (Zhang and Liu, 2015). Combined with the transcriptome data of this study, the expression of phenylpropanoid biosynthesis-related genes in patchouli was significantly downregulated under the treatment of p-HBA, and the expression of the phenylalanine ammonia lyase gene was significantly downregulated, corresponding to the decrease in PAL enzyme activity, with effects on plant secondary metabolism. The decrease in PAL was guaranteed to inhibit the metabolic flux of various branches of pathways from the oxalic acid pathway to the phenylalanine metabolic pathway and to hinder the downstream lignin and flavonoids metabolic pathways. These effects of decreased PAL expression lead to blockade of the core metabolic pathways in patchouli roots and inhibition of root growth.

**FIGURE 8:**
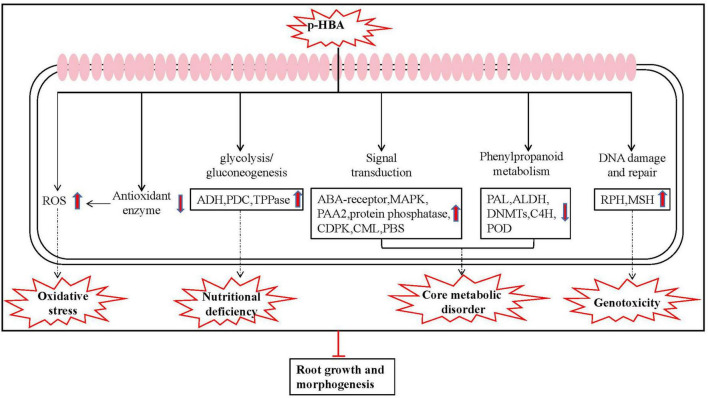
Schematic presentation of the specific response mechanism of roots to p-HBA stress in patchouli.

In summary, based on the above results, we deduced the mechanism underlying the p-HBA response in patchouli roots, as shown in Figure 8. After patchouli roots were subjected to p-HBA stress, the antioxidant enzyme protection system was damaged, resulting in increased free radicals and oxidative stress. Transcriptome analysis showed that p-HBA reduced the expression of genes related to antioxidant enzymes and increased DNA damage and genomic instability. p-HBA affected the phenylpropanoid metabolism pathway, resulting in inhibition of the downstream pathways, decreased synthesis of lignin and flavonoids, weakening of stress resistance in patchouli roots, and inhibition of root growth. These changes greatly affected the expression of genes encoding transcription factors, proteins involved in energy metabolism and other enzymes, which exacerbated metabolic dysfunction further disrupting homeostasis, and destroying root growth and development. With the extension of stress time, the stress response system of patchouli seedlings deteriorated or even failed.

## Conclusion

In summary, p-HBA treatment markedly retarded patchouli seedling growth with reduced shoot height, root development, and biomass. p-HBA supplementation gave rise to oxidative stress, reflected by increased ROS and MDA levels in patchouli roots. More importantly, exogenous application of p-HBA significantly activated the expression of genes related to ethanol dehydrogenase, pyruvate decarboxylase, Ca^2+^/calmodulin-dependent EF-Hand protein kinase, calcium-dependent protein kinase, and inhibited the expression of genes related to peroxidase, disease resistance protein RPM1, phenylalanine ammonia lyase. As a result, the root growth of plants was damaged and the toxic effect was intensified. This study provides valuable insights into the physiological and molecular response mechanisms of patchouli plants to autotoxicity. The information gathered here will provide an excellent resource to extensively explore autotoxicity stress resistance genes and metabolites and assist in plant breeding to alleviate continuous cropping obstacles.

## Data availability statement

The data presented in this study are deposited in the National Center of Biotechnology Information (NCBI) Sequence Read Archive (SRA) repository, accession number: PRJNA850618.

## Author contributions

YGW, WGY, and WPY designed the experiments. WPY, SJC, and XFL performed the experiments. WPY, GLY, TFB, and JY analyzed the data. WPY, SJC, and JFZ wrote the manuscript. All authors have read and approved the final manuscript.

## Footnotes

1
www.oebiotech.com
