# Supplementary material for: Combined physiological and transcriptome analysis revealed the response mechanism of Pogostemon cablin roots to p-hydroxybenzoic acid
Source: Front Plant Sci. 2022 Sep 26;13:980745. doi: 10.3389/fpls.2022.980745 (PMC9549242; doi:10.3389/fpls.2022.980745)
Supplement: Supplementary file 2 [file Data_Sheet_2.docx]

***Supplementary Materials***

**
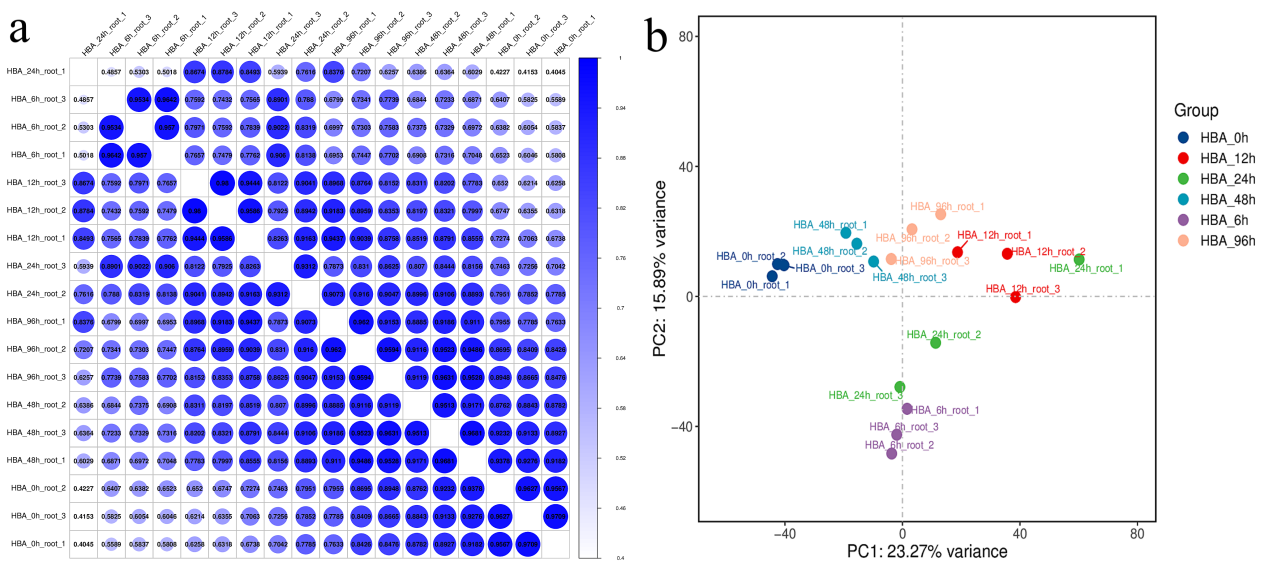
**

**Supplementary Figure 1. Correlation heat map (a) and Principal component analysis diagram (b)** **in transcriptomic profile of patchouli root samples.**

**
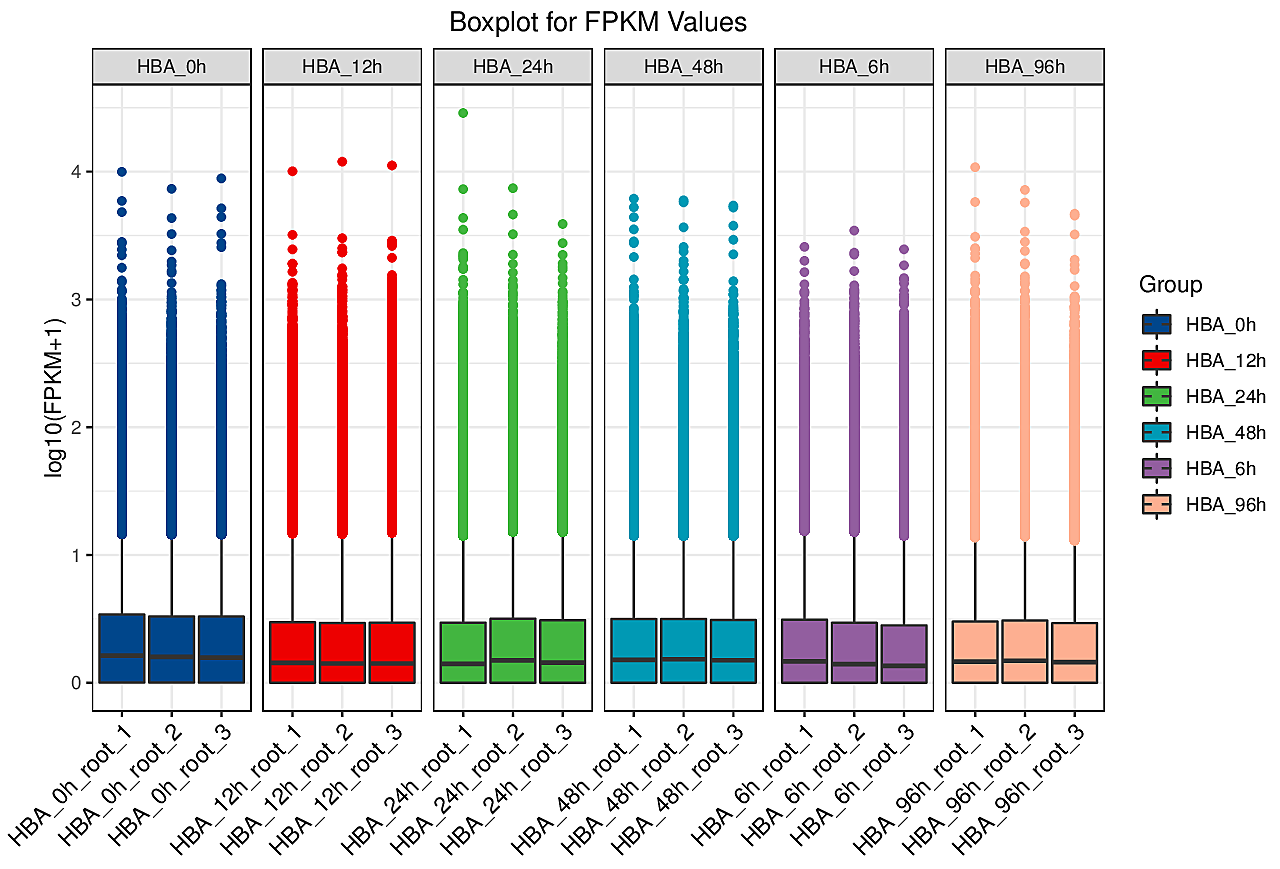
**

**Supplementary Figure 2. The fragments per kilobase of transcript per million mapped reads (FPKM) boxplot in the 18 libraries.**
